# Formulation, E-Beam Crosslinking, and Comprehensive Characterisation of Lavender Oil-Enriched Hydrogels

**DOI:** 10.3390/polym16223150

**Published:** 2024-11-12

**Authors:** Maria Demeter, Ion Călina, Anca Scărișoreanu, Monica R. Nemțanu, Mirela Brașoveanu, Marin Micutz, Marius Dumitru

**Affiliations:** 1 National Institute for Laser, Plasma and Radiation Physics, 409 Atomiștilor, 077125 Măgurele, Romania; maria.demeter@inflpr.ro (M.D.); monica.nemtanu@inflpr.ro (M.R.N.); mirela.brasoveanu@inflpr.ro (M.B.); marius.dumitru@inflpr.ro (M.D.); 2 Department of Physical Chemistry, University of Bucharest, 4-12 Regina Elisabeta Blvd, 030018 Bucharest, Romania; marin.micut@chimie.unibuc.ro

**Keywords:** e-beam irradiation, degradation, lavender oil, porous structure, structural stability, superabsorbent hydrogel

## Abstract

This study focused on the formulation, electron beam (e-beam) crosslinking, and characterisation of hydrogels enriched with lavender oil (LO) to enhance their structural and functional properties for biomedical applications. Stable hydrogels were synthesised using water-soluble polymers and suitable ratios of Tween 80 and Isopropyl alcohol (IPA) as surfactant and co-surfactant, respectively, via e-beam irradiation at doses up to 70 kGy. The most effective crosslinking was achieved with a radiation dose of 30 kGy, depending on the concentrations of surfactants and LO. LO-enriched hydrogels exhibited enhanced superabsorbent swelling (7700% to 18,000%) and faster equilibrium rates than the control hydrogel. Structural analysis revealed a flexible spongiform porous architecture with larger mesh sizes (156 nm to 246 nm) and adequate elastic moduli (130 to 308 Pa). Degradation tests aligned with swelling data, demonstrating a degradation rate of 12% after 35 days, indicating an appropriate balance of stability and degradation. These findings suggest that e-beam technology, in conjunction with LO and surfactant addition, can effectively tailor hydrogel properties for biomedical applications, making them promising candidates for further research in wound care, drug delivery systems, and other biological applications.

## 1. Introduction

Essential oils (EOs) are rich in bioactive compounds with well-known antimicrobial, antioxidant, and anti-inflammatory properties, making them ideal candidates for wound healing applications. Phytochemicals, such as terpenes, phenols, and alkaloids, which are present in EOs, promote faster recovery by preventing infections, reducing oxidative stress, and controlling inflammation [1]. Despite these benefits, the direct use of EOs in topical formulations remains challenging due to their sensitivity to environmental factors, such as air, light, humidity, and high temperature, which can cause rapid degradation of active compounds and reduced therapeutic efficacy [2]. Among EOs, lavender oil is a versatile oil used for wound healing, extracted from various species of the lavender plant, including *Lavandula angustifolia*, *Lavandula stoechas*, and *Lavandula latifolia*. LO primarily consists of two active compounds: linalool and linalyl acetate, which reduce inflammation, decrease water retention, and facilitate tissue repair [3].

Hydrogels, with their highly hydrophilic structure, offer significant potential as a matrix for bioactive compounds in wound healing due to their excellent absorption capacity, biocompatibility, and prolonged skin contact. However, their hydrophilic nature presents difficulties in directly incorporating hydrophobic molecules such as EOs [4]. Various approaches, including emulsification, fumigation, and encapsulation, have been explored to overcome these limitations, creating more stable EO-enriched hydrogel systems suitable for biomedical use [5].

Among various crosslinking techniques, e-beam crosslinking is regarded as a particularly advantageous method for hydrogel synthesis. Unlike chemical crosslinking, which often requires the addition of potentially toxic crosslinkers, e-beam technology offers a clean process that avoids the need for chemical initiators. This results in biocompatible and non-toxic hydrogels, a critical requirement for biomedical applications, such as wound dressings and drug delivery. Additionally, e-beam crosslinking allows for tailoring the hydrogel properties, such as swelling capacity, elasticity, and mechanical strength by adjusting the radiation dose [6,7,8]. Compared to other crosslinking approaches, which can sometimes degrade sensitive bioactive compounds, e-beam processing occurs at lower temperatures, reducing the risk of thermal degradation of EOs and other sensitive components. This makes e-beam crosslinking a suitable approach for developing EO-enriched hydrogels, ensuring both the integrity of the active compounds and the stability of the hydrogel matrix. 

Recent studies have demonstrated the successful incorporation of EOs into various hydrogel systems, with promising results, such as enhanced antimicrobial activity, controlled release, and improved wound healing performance. For instance, Rusu et al. [9] synthesised hybrid hydrogels by mixing poly(itaconic anhydride)-co-3,9-divinyl-2,4,8,10-tetraoxaspiro[5.5] undecane with sodium alginate, which was crosslinked using phytic acid, and then the optimal hydrogel composition was embedded with LO. These hydrogels exhibited improved antimicrobial properties against *S. aureus* and *C. albicans* due to LO addition, and the in vivo biocompatibility test indicated enhanced anti-inflammatory properties compared to the hydrogel without LO. Mahmood et al. [10] reported remarkable antioxidant activity of gellan gum hydrogels co-enriched with LO and ofloxacin, which showed the ability to reduce the harmful effects of free radicals. Another LO-based hydrogel showed efficiency in healing infected burn wounds and exhibited antibacterial properties, making it a promising dressing for treating drug-resistant infections [11].

Other studies [12] have focused on hydrogels composed of keratin and polyvinylpyrrolidone (PVP) synthesised using UV-crosslinking (400 W, λ = 254 nm) for 30 min and enhanced with 1% to 5% LO. The antibacterial tests performed on gram-positive and gram-negative bacteria demonstrated the antibacterial efficacity of these hydrogels, confirming their suitability for applications as wound dressings.

Despite these advances, to the best of our knowledge, no research to date has explored the use of e-beam crosslinking to create LO-enriched hydrogels based on PVP, carboxymethyl cellulose (CMC), and polyethylene oxide (PEO). This highlights one of the gaps in the literature, particularly in terms of the potential for e-beam radiation processing to enhance the structural and functional properties of hydrogels for biomedical applications.

This study proposes to address this gap by developing and characterising e-beam crosslinked hydrogels enriched with LO as an active ingredient. By utilising water-soluble polymers, such as PVP, CMC, and PEO, combined with proper surfactants and co-surfactants, this research has the potential to design stable, high-performance hydrogels with enhanced properties, such as swelling capacity, physiological stability, elasticity, and homogeneous structure. These formulations could offer a feasible alternative for expanding the application of LO-enriched hydrogels in medical, pharmaceutical, and cosmetic fields.

## 2. Materials and Methods

### 2.1. Materials

Polyvinylpyrrolidone (PVP, Mw = 1.3 × 10^6^ g/mol), sodium carboxymethyl cellulose (CMC, Mw = 2.5 × 10^5^ g/mol), polyethylene oxide (PEO, Mw = 3 × 10^5^ g/mol), and Tween 80 (Polysorbate 80, Mw = 7.9 × 10^4^ g/mol) were all purchased from Sigma Aldrich (St. Louis, MO, USA). Isopropyl alcohol (IPA, Mw = 60.1 g/mol, 99.8%) was obtained from Sedachim SRL (Târgoviște, Romania), and lavender oil (LO) was acquired from Hofigal Export–Import S.A Bucharest (Bucharest, Romania).

### 2.2. Synthesis of LO-Enriched Hydrogels via E-Beam Crosslinking 

First, PVP, CMC, and PEO stock aqueous polymer solutions were prepared in deionised water (DI-water). The initial polymeric composition (IPC), prepared from stock solutions and expressed for a volume of 100 mL, consisted of 3.1% PVP, 0.65% CMC, 0.21% PEO, and DI-water to complete the volume. IPC exhibited a pH of 6.5, was transparent, and had an apparent viscosity of 107 mPa·s.

For the emulsion base (designated as Smix), three distinct mixtures were formulated using varying ratios of Tween 80 and IPA: 1:4 (Smix 3), 2:1 (Smix 4), and 3:1 (Smix 5). Each Smix was then combined with LO in specific ratios to create 11 distinct formulations to identify the most stable emulsions. The LO was dispersed into the Smix following mass ratios of 1:9, 1:8, 1:7, 1:6, 1:5, 1:4, 1:3.5, 1:3, 1:2.3, 1:1.5, and 1:1. Thus, the LO-pre-hydrogels were prepared using a titration method involving a homogeneous mixture of LO, surfactant, co-surfactant, and IPC, achieved through mechanical stirring at room temperature (22 ± 1 °C). The samples were labelled with a number corresponding to the mass ratio of LO to Smix and the corresponding number of the Smix mixture. 

Table 1 shows data on phase separation observed after 48 h and 60 days for LO-pre-hydrogels. The control pre-hydrogel (without LO) was irradiated at 30 kGy. In the case of mixtures derived from Smix 3, the initial irradiation dose did not facilitate crosslinking, prompting an increase in the irradiation dose to 70 kGy. Conversely, the emulsions formulated using Smix 4 and Smix 5 successfully underwent crosslinking at the initial dose of 30 kGy. To produce homogeneous hydrogels, the final solutions comprising Smix, LO, and the polymeric mixture were degassed before irradiation through sequential centrifugation at 800 rpm for 20 min and ultrasonication at 37 Hz (degassing function). Before irradiation, the emulsions were stored in cylindrical plastic vessels designed to be airtight.

All irradiations were conducted at the Electron Accelerators Laboratory (INFLPR, Măgurele, Romania), using a linear electron accelerator (mean energy of 5.5 MeV) at doses between 30 and 70 kGy, with a dose rate of 5 kGy/min. The nominal irradiation dose and dose rate were measured using calibrated graphite calorimeters against standard reference alanine dosimeters, with a measurement uncertainty of ±5%.

The preliminary selection of both pre-hydrogels and LO-enriched hydrogels was based on several criteria, including phase separation of specific non-irradiated samples at 48 h and 60 days post-preparation, irradiation dose, viscometry analysis, optical transparency, colour analysis, gel fraction, swelling characteristics, and degradation.

### 2.3. Viscosity Analysis

The shear stress, *τ*, of the pre-hydrogels was assessed using the VT® 550 rotational rheometer (Haake GmbH, Karlsruhe, Germany), equipped with a coaxial cylinder NV. The measurements were conducted at ambient temperature (22 ± 1 °C) over various shear rates, $\dot{\gamma }$, ranging from 5 to 2164 s^−1^. The Ostwald–de Waele rheological model (Equation (1)) was used for fitting shear stress data and then to calculate the apparent viscosity ($\eta_{a}$) of the samples at $\dot{\gamma }$ = 200 s^–1^ and 22 °C.

(1) $$\tau =k\cdot {\dot{\gamma }}^{n-1},$$

where: *τ*—shear stress [Pa], *k*—fluid consistency coefficient [mPa∙s^n^], *n*—flow behaviour index (dimensionless), and $\dot{\gamma }$—shear rate [s^−1^].

### 2.4. Optical Transparency

UV–Vis transmittance spectra for IPC and LO-pre-hydrogels were measured using a spectrophotometer Jenway 6850 (Jenway, Bibby Scientific Ltd., Stone, UK) at wavelengths ranging from 190 to 800 nm. The measurements were conducted one-day post-preparation and again after approximately 80 days. To determine the transmittance (T%), 200 µL of the sample was diluted in 5 mL of DI-water before analysis.

### 2.5. Colour Characterisation of Pre-Hydrogels

Spectro-colorimetric measurements were conducted on IPC and LO-pre-hydrogels using a Cary 100 Bio spectrophotometer (Varian, Inc., Walnut Creek, CA, USA). The measurements were performed in transmittance (T%) within the visible spectrum (360–830 nm) at ambient temperature (22 ± 1 °C) for the standard illuminant D65 (natural light source), with a 10° observer angle. The colour changes of the samples were assessed using two colour coordinate systems: CIE L*a*b* and CIE L*C*h^0^. The characteristics of colour were expressed as CIELAB L* (lightness ranging from 0 for black to 100 for white), a* (red–green coordinate where +a* indicates red and −a* indicates green), and b* (yellow–blue coordinate where +b* indicates yellow, −b* indicates blue). In the CIELCH system, the characteristics included L* (lightness), C* (chroma), and *h*^0^ (hue). The colour of the pre-hydrogel mixture served as a reference to determine the overall colour difference, ΔE_ab_, of each sample.

### 2.6. Gel Fraction and Swelling Analysis

Hydrogels with and without LO were dehydrated in an oven at temperatures lower than 30 °C for 72 h. Following this, they were extracted with DI-water at ambient temperature (22 ± 1 °C) for 48 h and then dried under the same conditions for another 72 h until a constant weight was achieved. The gel fraction of the cross-linked hydrogels was quantified gravimetrically, following the methodology described by Nagasawa et al. [13].

The swelling characteristics of the hydrogels were assessed in DI-water and several pH buffer solutions, including citrate buffer at pH 5.4, saline phosphate buffer at pH 7.4, and bicarbonate buffer at pH 9.4. These tests were conducted at 37 °C using a thermostatic oven. At designated time intervals, the hydrogels were extracted from the swelling medium, gently wiped with filter paper to eliminate excess solvent from the surface, and subsequently weighed. The swelling degree (*SD*%) was calculated based on the weight change, following a previous methodology [14].

### 2.7. Degradation Study

The degradation of LO-enriched hydrogels was evaluated under conditions simulating the physiological environment, specifically using phosphate-buffered saline (PBS) (pH = 7.4, 37 °C) over 65 days. The weight loss was measured gravimetrically at specified intervals throughout the study. The degradation percentage (%) for each time point was calculated using the following formula [15].

(2) $$Weight\;loss\;\left(\%\right)=\frac{w_{i}-w_{f}}{w_{f}}\times 100\;,$$

where *w_i_*—initial weight of the sample, and *w_f_*—final weight of the sample.

### 2.8. Fourier Transform Infrared Spectroscopy (FTIR) Analysis

The structural changes induced by e-beam irradiation in all hydrogel samples were analyzed using ATR–FTIR spectroscopy. FTIR spectra were obtained from freeze-dried samples using a Perkin Elmer Spectrum 100 instrument (Perkin Elmer, Waltham, MA, USA) with a diamond crystal and operating in ATR mode. The spectral range analyzed was from 4000 to 650 cm^−1^, with a resolution of 4 cm^−1^. For each sample, FTIR spectra were acquired through 30 scans. 

### 2.9. Rheological and Network Structural Properties

Rheological investigation of hydrogels was performed using a Micro Fourier Rheometer MFR 2100 (GBC, Australia). The viscoelastic parameters, specifically the elastic modulus (*G′*) and viscous modulus (*G″*), were derived from the Fourier analysis of the force signal collected at 400 discrete frequencies simultaneously. The measurements were conducted at a constant temperature of 23 °C, with a gap between plates of 400 μm, a displacement amplitude of 0.03 μm, a frequency range of 0.005 to 2.000 Hz, and an equilibration period of 20 min for each isothermal experiment, comprising 30 scans/reograms. The behaviour and amplitude of the *G′* and *G″* moduli, alongside the flow stresses and the applied force (oscillation frequency), were theoretically modelled to obtain information about the gel structure. According to the rubber elasticity theory, the *G′* and *G″* moduli are multiplied by the polymer volume fraction in the swollen state ${\left(\nu_{2,s}\right)}^{1/3}$ and the post-irradiation polymer volume fraction ${\left(\nu_{2,r}\right)}^{2/3}$ (after crosslinking), leading to the calculation of the crosslinking density ($V_{e}$) of the system and the molecular weight between two consecutive crosslinking points ($M_{C})$. The mathematical equations that describe the previous parameters can be found in the relevant literature [16,17]. 

### 2.10. Scanning Electron Microscopy (SEM)

The morphology of the hydrogels was examined with a 20 kV scanning electron microscope FEI Inspect S model (FEI Co. Ltd., Hillsboro, OR, USA). Hydrogel samples, swollen to equilibrium in DI-water, were freeze-dried before SEM analysis. The cross-sections of the freeze-dried hydrogels were examined after gold coating. The SEM images were captured at magnifications of 100×, 250×, 20,000×, and 40,000× to provide detailed insights into hydrogel structure.

### 2.11. Statistical Analysis

The experimental results are presented as mean values ± standard deviation, derived from three measurements. The data were evaluated with analysis of variance (ANOVA) accompanied by the Fisher LSD post hoc test to identify statistical differences. A probability value of *p* ≤ 0.05 was considered to indicate statistical significance. 

## 3. Results and Discussion

### 3.1. Pre-Hydrogel Flow Behaviour and Viscosity

Rheological properties, particularly flow behaviour and viscosity, are important for understanding the performance and stability of pre-hydrogels before they undergo cross-linking. These properties can influence the ease of processing and handling during formulation. The rheological characteristics of the IPC and LO-pre-hydrogels were evaluated to assess sample stability. Samples with visible phase separation were excluded from viscosity tests to ensure consistency in the tested formulations and reduce errors caused by phase instability. 

The experimental results indicated non-Newtonian flow behaviour for all samples, as evidenced by the non-constant ratio of shear stress (τ) to shear rate (γ). Applying the Ostwald de Waele model to all samples resulted in high correlation coefficients (r = 0.9987 to 0.9996), confirming a good fit for the data. Tables S1–S3 provide the resulting rheological parameters, n (flow behaviour index) and k (fluid consistency coefficient). The flow behaviour index (n < 1 for all samples) confirmed the shear-thinning behaviour, where lower values of n correspond to a greater degree of shear-thinning [18]. This behaviour might be due to structural changes, such as pronounced elongation, network collapse, and deformation of emulsion droplets under increasing shear stress [19]. Among the tested samples, the formulations containing Smix 3 (sample 3-3) exhibited the highest flow behaviour index, indicating a more stable flow under applied shear stress. Enrichment of the IPC with LO generally reduced the fluid consistency coefficient (*p* ≤ 0.05), suggesting that the LO-pre-hydrogels exhibited easier flow. This could be attributed to the structural influence of LO:Smix on the polymer network, which slightly reduced the resistance to flow. However, formulations containing Smix 4 and Smix 5 exhibited generally a smaller reduction in the fluid consistency coefficient than those with Smix 3.

The apparent viscosity results consistently revealed lower values (*p* ≤ 0.05) for LO-pre-hydrogels compared to the IPC (Tables S1–S3), indicating that the addition of LO also improved flow. This trend was particularly evident in formulations containing Smix 3, where high LO:Smix ratios were associated with a substantial drop in viscosity. However, in the cases of LO:Smix ratios of 1:9, 1:8, and 1:6, an increase in viscosity was observed as the proportion of surfactant increased. This may be attributed to enhanced resistance to droplet movement within the emulsion, resulting in increased viscosity.

The rheological findings highlight the significant influence of LO enrichment and specific Smix compositions on the flow behaviour and viscosity of pre-hydrogels. Formulations containing Smix 4 and Smix 5 exhibited lower viscosity loss, generally not exceeding 6%, compared to the IPC, and maintained comparable flow behaviour, indicating good stability.

### 3.2. Optical Transparency

Optical transparency is a key criterion that reflects the stability of an emulsion, as it indicates the uniformity of the dispersion and the absence of phase separation or aggregation over time. To assess the formulation’s stability, UV-Vis transmittance spectra of both the IPC and LO-pre-hydrogels were measured one-day post-preparation and again after approximately 80 days. As shown in Figure S1 the initial IPC transparency, expressed as T%, was approximately 96%.

LO-pre-hydrogels prepared with Smix 4 and Smix 5 formulations exhibited high optical transparency, exceeding 97%, except formulations (9-5), which had a transparency of 86%. A high transparency level (>85%) indicated the absence of phase separation, reflecting the stability of the formulation. Conversely, the optical transparency of the LO-pre-hydrogels formulated with Smix 3 (sample 8-3) showed a marked reduction to 48%, indicating a potential stability issue, likely due to phase separation or changes in the internal structure of the emulsion. Notably, the LO-pre-hydrogels with higher concentrations of LO and Tween 80 (Smix 4 and Smix 5) demonstrated good stability, maintaining their high transparency levels even after 80 days of storage. The ability to retain optical transparency over extended periods indicates that these formulations resist physical changes, such as phase separation or droplet aggregation, which is critical for long-term stability in practical applications.

### 3.3. Colour Characterisation of Pre-Hydrogels

A detailed analysis of the pre-hydrogel colour properties, including variations in CIE L*a*b* and CIE L*C*h^0^ coordinates and their implications for sample stability, is presented in the Supplementary File.

### 3.4. Gel Fraction and Swelling Properties

**Figure 1:**
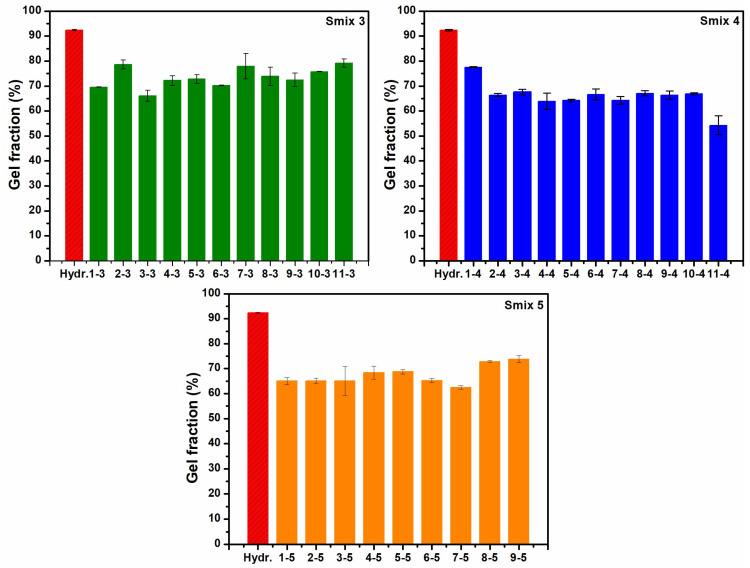
Gel fraction of control hydrogel and LO-enriched hydrogels.

All three LO-enriched hydrogel types were transparent, maintained structural integrity, and exhibited non-adhesive properties during handling. Figure 1 illustrates the gel fraction as a function of the radiation dose. The gel fraction for the control hydrogel (without LO) was 92% while, for the LO-hydrogels, gel fractions varied from 66% to 80% for Smix 3, 54% to 78% for Smix 4, and 62% to 73% for Smix 5, depending on composition. Gel fractions exceeding 40% suggest that the polymers retained their integrity during irradiation, indicating the effectiveness of the crosslinking process [20]. Although Smix 3 hydrogels exhibited higher gel fractions, they required irradiation doses of up to 70 kGy to complete crosslinking.

**Figure 2:**
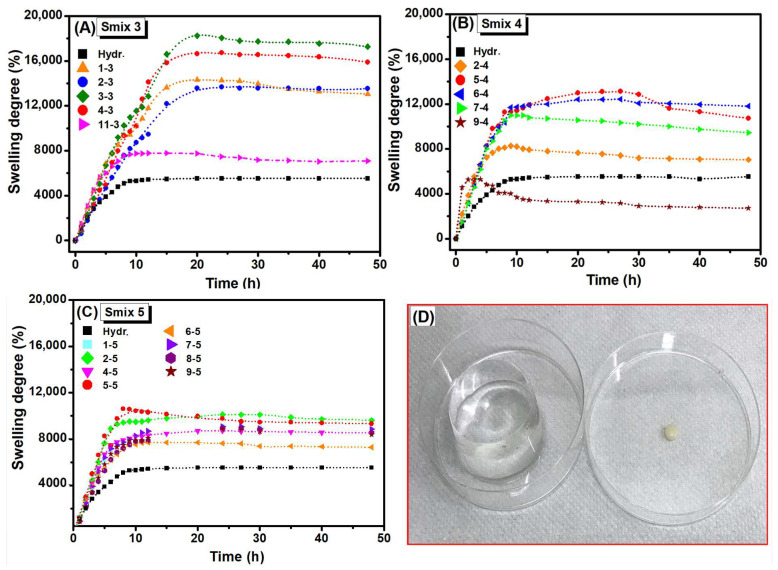
*SD*% at the equilibrium of LO-enriched hydrogels: (**A**) Smix 3; (**B**) Smix 4; (**C**) Smix 5, and (**D**) control hydrogel in the dry and swollen state.

Figure 2 shows the swelling degree (*SD*%) of the LO-enriched hydrogels in DI-water as a function of composition. The control hydrogel exhibited an *SD*% of approximately 5500%, reaching equilibrium after 15 h of immersion. In contrast, LO-hydrogels exhibited significantly higher swelling, approximately two to three times greater than the control hydrogel. Moreover, after 20 h, Smix 3 hydrogels reached swelling equilibrium with values ranging from 7700% to 18,000%. Smix 4 and Smix 5 hydrogels achieved equilibrium more quickly, after approximately 10 h, with a reduced *SD*% between 7700% and 11,300%.

Notably, a reduction in *SD*% was observed in all hydrogels with higher LO concentrations. For hydrogels with an equal LO:Smix ratio (i.e., formulation 11-3), the swelling was reduced to 8000%, yet it remained higher than that of the initial crosslinked hydrogel. Smix 3 formulations (samples 3-3 and 4-3), corresponding to LO:Smix ratios of 1:7 and 1:6, exhibited a greater swelling. The swelling degree varied with both LO and Tween 80 contents. Specifically, Smix 4 formulations (ratios of 1:5, 1:4, and 1:3) demonstrated enhanced water affinity. However, formulations with a 1:2.3 ratio exhibited lower swelling than the control hydrogel, indicating that higher LO concentrations might favour an increased crosslinking density. Smix 5 hydrogels showed reduced swelling compared to Smix 3 and Smix 4, yet most formulations remained stable in swelling media despite higher LO and surfactant concentrations. The results indicate that, in Smix 3 formulations, an increased concentration of IPA promoted swelling but reduced mechanical strength. Conversely, a higher concentration of Tween 80 enhanced the solubilization capacity of the hydrophobic component (LO) within the aqueous polymer matrix, facilitating moderate crosslinking and leading to hydrogels with stable mechanical structures, as confirmed by subsequent rheological analyses. The substantial hydrogel swelling (9000% to 11,800%) can be attributed to Tween 80’s ability to promote porous architecture by reducing crosslinking density [21].

**Figure 3:**
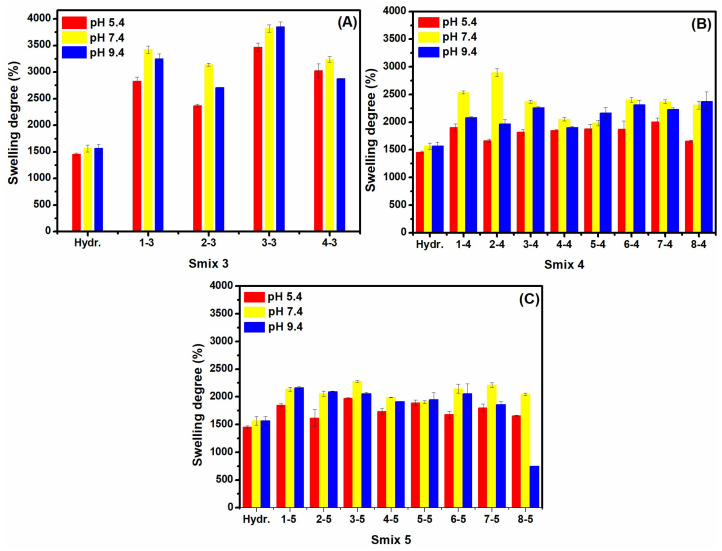
The equilibrium swelling degree of LO-enriched hydrogels in different pH conditions: (**A**) Smix 3; (**B**) Smix 4, and (**C**) Smix 5.

Figure 3 illustrates the equilibrium *SD*% of LO-hydrogels at pH values of 5.4, 7.4, and 9.4, and a constant temperature of 37 °C. These conditions mimic the environment of infected wounds [22]. The control hydrogel generally had lower swelling than LO-hydrogels across all pH levels. At physiological pH (7.4), Smix 3 hydrogels exhibited greater swelling than Smix 4 and Smix 5, indicating a lower crosslinking degree. In contrast, Smix 4 and Smix 5 hydrogels demonstrated reduced swelling, signifying an increased crosslinking, as water diffusion was more restricted. Hydrogel swelling behaviour is primarily influenced by the available free volume within the polymer matrix, due to the total number of ionizable functional groups (such as NH₄⁺ and COO^−^), and the extent of crosslinking [23]. In an acidic medium, Smix 3 hydrogels also showed superior swelling compared to Smix 4 and Smix 5, likely due to the high concentration of ionizable groups (COO^−^ and HO^−^) in both the polymer and co-surfactant molecules, which enhanced hydrophilicity. This greater swelling suggests reduced crosslinking, despite Smix 3 requiring a radiation dose of 70 kGy for synthesis. In radiation processing, higher doses typically enhance crosslinking in synthetic polymers, whereas natural polymers often undergo chain scission at high doses [24]. At acidic pH, repulsion between neighbouring COO^−^ groups can increase the available space between hydrogel chains or break the hydrogen bonds, enhancing liquid absorption. At basic pH, the complete ionization of carboxyl groups and the breaking of hydrogen bonds lead to more pronounced swelling. This behaviour was particularly evident in Smix 3 hydrogels, demonstrating a lower crosslinking degree than that of Smix 4 and Smix 5 [25].

### 3.5. Hydrogel Degradation

**Figure 4:**
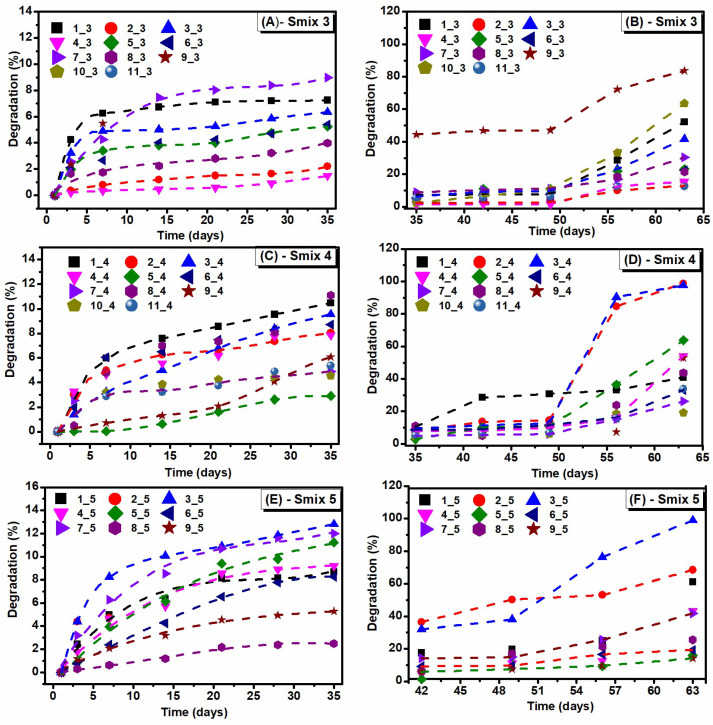
Degradation in PBS at 37 °C of LO-enriched hydrogels: (**A**,**B**)—Smix 3; (**C**,**D**)—Smix 4, and (**E**,**F**)—Smix 5.

The degradation behaviour of the LO-hydrogels is illustrated in Figure 4. During the first 7 days, an average weight loss of ~7% was observed across all three hydrogel formulations. Between days 7 and 35, the weight loss was up to 12%. After 50 days, a significant weight loss was observed for most hydrogels and, by day 63, some formulations had degraded completely under conditions simulating a physiological environment (PBS at 37 °C). In contrast, the control hydrogel exhibited minimal degradation, with a rate of less than 2%.

As demonstrated in the swelling experiments, LO-hydrogels exhibited significant swelling capacities associated with varying degradation rates. Thus, Smix 3 formulations showed an SD of ~19,000%, with degradation exceeding 50%. Smix 4 hydrogels had swelling of ~16,700% and 33% degradation, and Smix 5 hydrogels exhibited an SD of ~14,000% with degradation of 43% rate. The relationship between swelling and degradation is essential because the degree of swelling reflects the ability of the hydrogel to absorb fluids, and degradation indicates its long-term stability or degradation in environmental conditions. Hydrogels with higher swelling capacities, such as those in the Smix 3 formulations, typically had a lower crosslinking degree, accelerating both fluid absorption and faster degradation. Conversely, lower swelling capacities in Smix 4 and Smix 5 hydrogels may indicate a dense polymer network or higher crosslinking density, resulting in slower degradation rates. A controlled and incremental degradation profile is particularly advantageous for hydrogels used as wet dressings on the skin, as these materials are less likely to degrade rapidly upon contact with human tissue. This slower degradation can reduce the need for frequent dressing changes while ensuring sustained exudate absorption [26]. Therefore, hydrogels with concomitantly substantial swelling capacity and moderate degradation rates may be suitable for maintaining wound moisture and structural stability over time. Hydrogels with significant swelling and degradation rates below 50% were selected for further investigations because these characteristics indicate a polymeric matrix with enhanced stability in structure. The bacterial cellulose/PVP/calcium phosphate hydrogels showed a significant decrease in weight loss of 42–52% after 7 days in physiological solution [27]. The rapid weight loss was due to hydrophilic polymers, such as bacterial cellulose and PVP, which are biodegradable. Roy et al. [28] found that PVP-CMC hydrogels showed a weight loss of approximately 38% within 8 weeks.

### 3.6. ATR–FTIR

**Figure 5:**
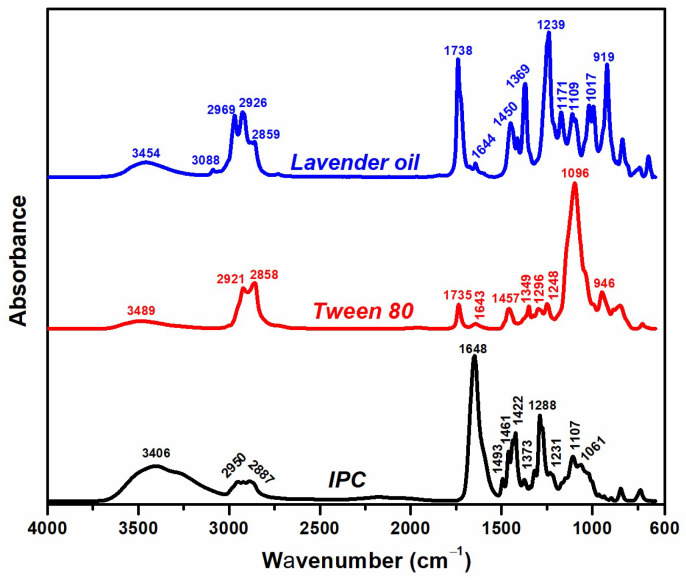
FTIR spectra of lavender oil, Tween 80, and IPC.

Figure 5 presents the FTIR spectra of LO and Tween 80, the primary components of the LO-enriched pre-hydrogels, along with the initial polymer composition (IPC). The absorption bands of LO include a peak at 3454 cm^−1^, indicating O-H stretching vibrations, and bands from 2969 to 2859 cm^−1^, associated with C-H and CH_2_ groups. The band at 1738 cm^−1^ represents C=O stretching vibrations, while the band at 1644 cm^−1^ is characteristic of C=C bonds. Additional bands at 1450 cm^−1^ and 1369 cm^−1^ correspond to C-H bending vibrations, and those at 1239, 1171, 1109, and 1017 cm^−1^ are linked to the C-O group vibrations [29]. The spectrum of Tween 80 exhibited a peak at 3489 cm^−1^ (O-H stretching), bands between 2921 and 2858 cm^−1^ (C-H and CH_2_ stretching), a peak at 1735 cm^−1^ for C=O stretching, and peaks at 1457, 1349, 1248, and 946 cm^−1^ for C-H and CH_2_ bending vibrations, as well as a band at 1096 cm^−1^ for C-O stretching [30]. The FTIR spectrum of the IPC showed major peaks at 3406 cm^−1^ (O-H stretching), 2950–2887 cm^−1^ (C-H and CH_2_ stretching), 1648 cm^−1^ (C=O and C-N stretching), and additional bands at 1461, 1422, 1373, and 1288 cm^−1^, attributed to the deformation vibrations of CH_2_ and O-H groups. The C-O-C stretching vibrations appeared at 1107 and 1061 cm^−1^ [31].

**Figure 6:**
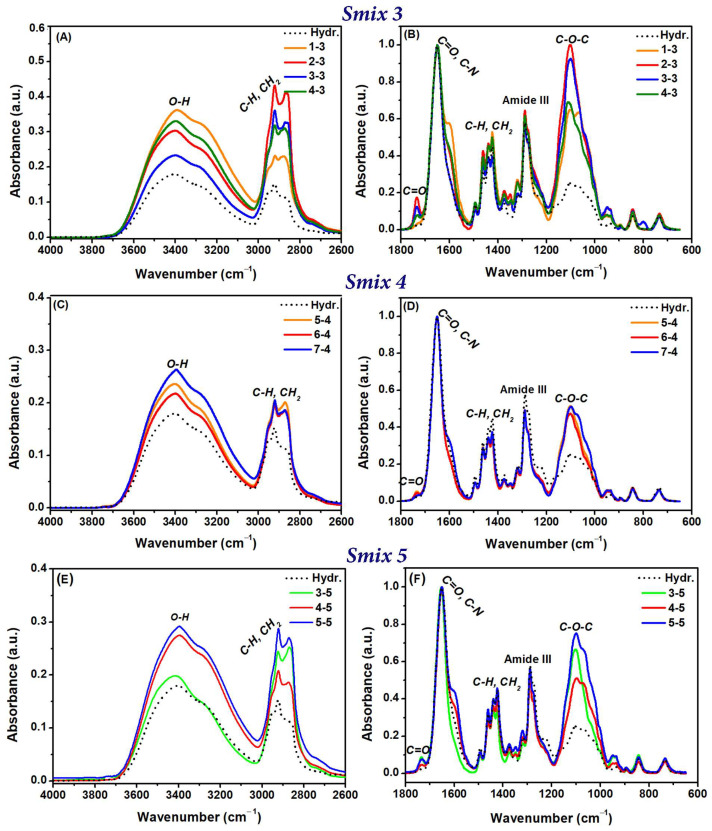
FTIR spectra for LO-enriched hydrogels: (**A**,**C**,**E**)—spectral range from 4000 to 2600 cm^−1^ for Smix 3, Smix 4, and Smix 5, respectively; (**B**,**D**,**F**)—spectral range from 1800 to 650 cm^−1^ for Smix 3, Smix 4, and Smix 5, respectively.

The FTIR spectra of the control and LO-enriched hydrogels are depicted in Figure 6. The control hydrogel, irradiated at a dose of 30 kGy, exhibited characteristic peaks at 3388 cm^−1^ (O-H groups), 2918 cm^−1^ (C-H and CH_2_ stretching vibrations typical of PVP, CMC, and PEO), and 1651 cm^−1^ (C=O stretching of PVP). Peaks at 1461, 1438, and 1423 cm^−1^ corresponded to CH_2_ bond deformations in PVP and CMC, while the peak at 1283 cm^−1^ was associated with amide III of PVP. Additional peaks at 1231 cm^−1^ and 1085 cm^−1^ corresponded to C-O and C-O-C groups, respectively, characteristic of PEO [32]. All LO-hydrogels showed a notable enhancement in band intensity in the 3700–2600 cm^−1^ region. In LO-hydrogels containing Smix 3, formulation 1-3 had the highest O-H band intensity, increasing by 20% compared to formulation 2-3, which showed a greater increase (~30%) in the C-H and CH_2_ groups. 

Additionally, a band shift from 3356 cm^−1^ to 3374 cm^−1^ was observed in the LO-hydrogels, suggesting an alteration in hydrogen bonding patterns. For Smix 4 and Smix 5 formulations, a proportional increase in the intensities of the O-H, C-H, and CH_2_ bands in the 3700–3000 cm^−1^ range was noted with increasing LO content. For instance, the O-H intensity in Smix 5 increased by 5% compared to Smix 4, where the C-H band intensity increased by 10%. Moreover, the CH_2_ peaks shifted to higher wavenumbers, reaching 2923 cm^−1^, while the C=O band at 1734 cm^−1^ remained unchanged. The slight shift of the C-O-C band to higher wavenumbers (from 1085 to 1103 cm^−1^), combined with an intensity increase of ~2.8 times (i.e., formulation 5-5), was also noted. The increased intensity of the C-O-C peak could be correlated with the extension of the polymer chain, likely due to the higher concentration of Tween 80. This chain extension enhances the ability of hydrogels to absorb water while maintaining mechanical integrity [33], which is essential for applications in wound dressings, where both swelling and stability are critical [34]. The presence of LO in the hydrogel matrix was confirmed by the appearance of a new band at 1734 cm^−1^, indicative of C=O groups [35]. The observed variations in band positions and intensity, particularly in C=O, C-O-C, and C-H bands, indicate stronger molecular interactions among the formulation components, likely due to crosslinking, which may support better polymer rearrangement [36]. These spectral changes can be associated with mechanical properties and hydrogel degradation behaviour.

### 3.7. Rheological Behaviour

**Figure 7:**
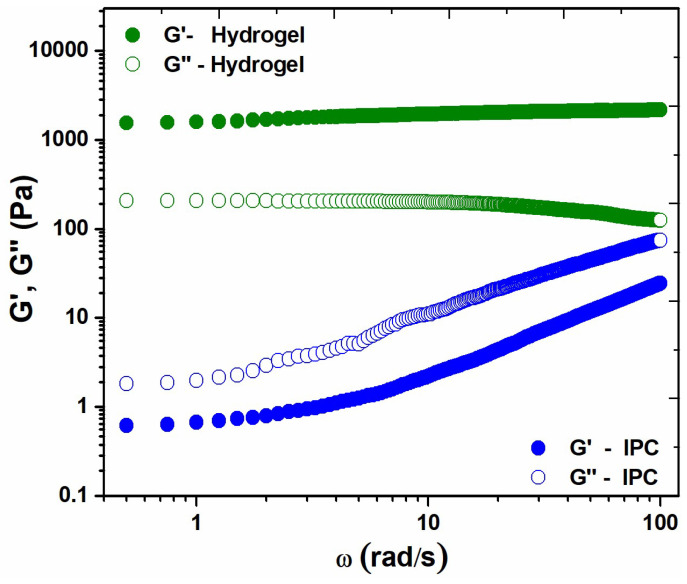
Elastic modulus (*G′*) and viscous modulus (*G″*) for (non-irradiated) IPC and corresponding crosslinked hydrogel.

The IPC demonstrated predominantly viscous behaviour, as indicated by *G″* > *G′*, typical of viscous liquids. Post-irradiation, *G′* of the control hydrogel increased significantly, maintaining a linear relationship over the frequency range and exceeding *G″*, with a value of 2169 Pa (Figure 7). *G′* > *G″* indicates that the hydrogel has a predominantly elastic behaviour, characteristic of solid-like materials, suggesting the formation of a permanent network structure due to crosslinking induced by e-beam irradiation [37].

**Figure 8:**
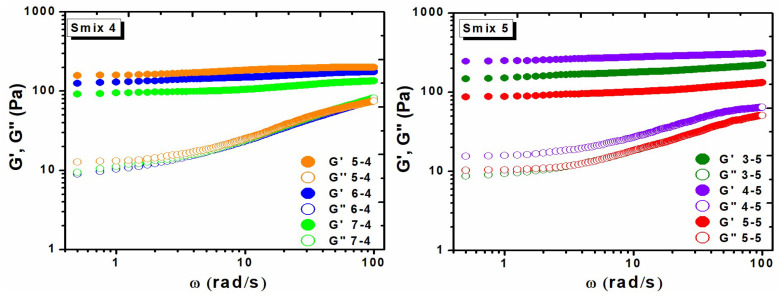
Elastic modulus (*G′*) and viscous modulus (*G″*) for the LO-enriched hydrogels of Smix 4 and Smix 5 formulations.

Figure 8 illustrates the relevant variation of *G′* and *G″* as a function of angular frequency ω for the LO-hydrogels corresponding to the Smix 4 and Smix 5 formulations. Their elastic modulus *G′* also exhibited a linear increase across the frequency domain, with an increase of at least two orders of magnitude relative to the *G″*, confirming the effective crosslinking and formation of LO-hydrogels. The magnitude of *G′* varied depending on the formulation, though it was notably lower than that of the control hydrogel, which experienced a significant decrease in *G′* value. This decrease may be related to the inclusion of LO and surfactants, which alter the structural rigidity of the hydrogel matrix. *G′* values ranged from 134 to 200 Pa for Smix 4 and 130 to 308 Pa for Smix 5. The variation in these values can be attributed to the compositional of surfactants and LO. Thus, Smix 4 hydrogels exhibited a lower *G′* but enhanced swelling capacity, while Smix 5 hydrogels had higher structural stability, as indicated by an increased *G′* and reduced swelling. Notably, these two hydrogel formulations included increased amounts of surfactant (Tween 80) compared to Smix 3. In particular, Smix 4 contained a lower concentration of Tween 80 and higher levels of LO than Smix 5, which had an increased concentration of Tween 80 and a reduced amount of LO. The reduction in *G′* is likely due to the increased concentration of Tween 80, which diminishes crosslinking density and promotes the formation of more flexible, porous and superabsorbent networks, when incorporated into hydrogel matrix [21]. In formulations 5-4, 6-4, and 7-4, the magnitude of *G′* decreases proportionally with increasing LO concentration, whereas formulation of Smix 5 exhibited a higher G’ modulus due to a lower LO content. In addition, previous studies where hydrogels were enriched with various essential oils (eucalyptus, ginger, and cumin) also showed reductions in *G′* compared to hydrogels without essential oils. For instance, the *G′* values for eucalyptus, ginger, and cumin oil-enriched hydrogels were 67.8 Pa, 124 Pa, and 217 Pa, respectively. This reduction in *G′* suggests that including essential oils may lead to structural changes in the hydrogel matrix, reducing the rigidity and increasing flexibility, likely due to the crosslinking network stability [38]. Thus, variations in concentrations in Tween 80 (surfactant) and LO could significantly influence the hydrogel’s mechanical properties by modulating the crosslinking efficiency and molecular interactions within the network, which promoted a denser crosslinked structure.

### 3.8. Network Structure

The analysis of the network parameters, including density (*ρ*), molecular weight between crosslinks (M_c_), crosslinking density (V_e_), and mesh size (*ξ*), revealed substantial variations in the macromolecular structures of the hydrogels, depending on their composition. Relevant results for LO-hydrogels containing Smix 4 and Smix 5 formulations are presented in Table 2. 

**Figure 9:**
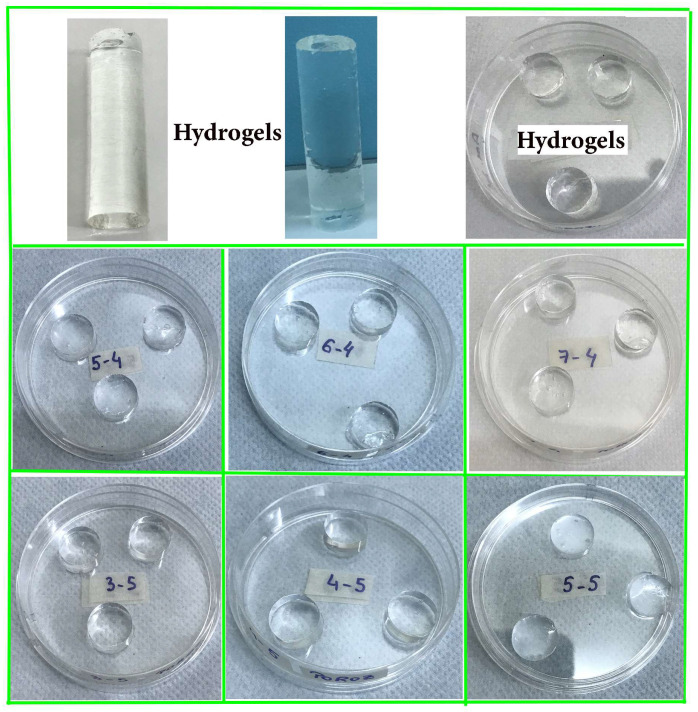
Optical images of LO-enriched hydrogels after e-beam cross-linking.

LO-hydrogels exhibited a significantly larger mesh size (*ξ*) than the control hydrogel, which showed a denser macromolecular network. The values of *ρ*, M_c_, and V_e_ for LO-hydrogels suggest that e-beam irradiation successfully produced hydrogels with substantial mesh sizes. The increased *ξ* and M_c_ found in LO-hydrogels indicate that the essential oil (LO) and surfactant (Tween 80) influence the crosslinking density, leading to more porous hydrogel structures. This phenomenon aligns with findings from swelling studies, which demonstrated that LO-hydrogels exhibited considerable swelling capacities, ranging from 8000% to 12,000% for both Smix 4 and Smix 5 formulations, due to their permanent network structure. Such behaviour is advantageous for applications requiring high fluid absorption, including wound dressings. Figure 9 shows the optical images of the investigated hydrogels upon e-beam irradiation.

These findings suggest that the variation in concentrations of essential oil and surfactant played a critical role in adjusting the network properties of the hydrogels. 

### 3.9. SEM Morphology

**Figure 10:**
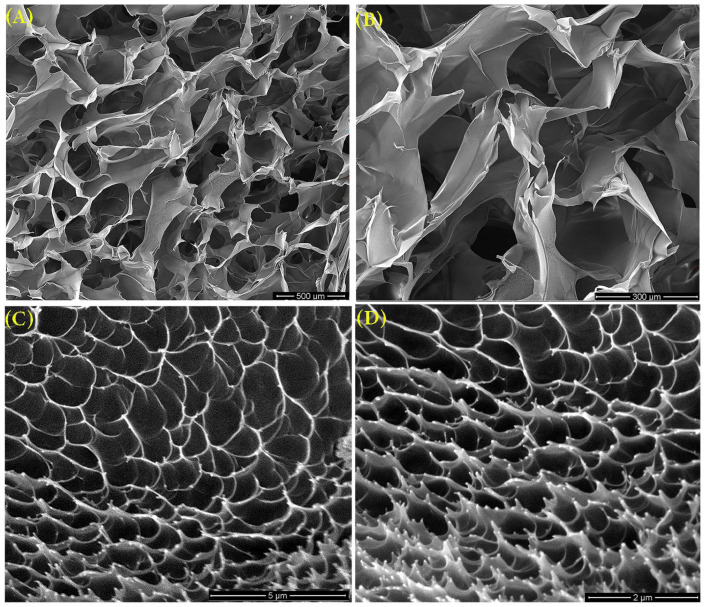
SEM images of the control hydrogel at magnifications: (**A**) 100×, (**B**) 250×, (**C**) 20,000×, and (**D**) 40,000×.

The structural analysis of the control hydrogel revealed significant porosity characterised by large and irregularly distributed pores (Figure 10). The internal architecture of this hydrogel exhibited notable variation (Figure 10C,D), showing a more compact and denser network. This compactness is primarily attributed to the enhanced effects of radiation crosslinking [39]. These observations are further supported by a high degree of crosslinking and a correspondingly reduced mesh size. 

**Figure 11:**
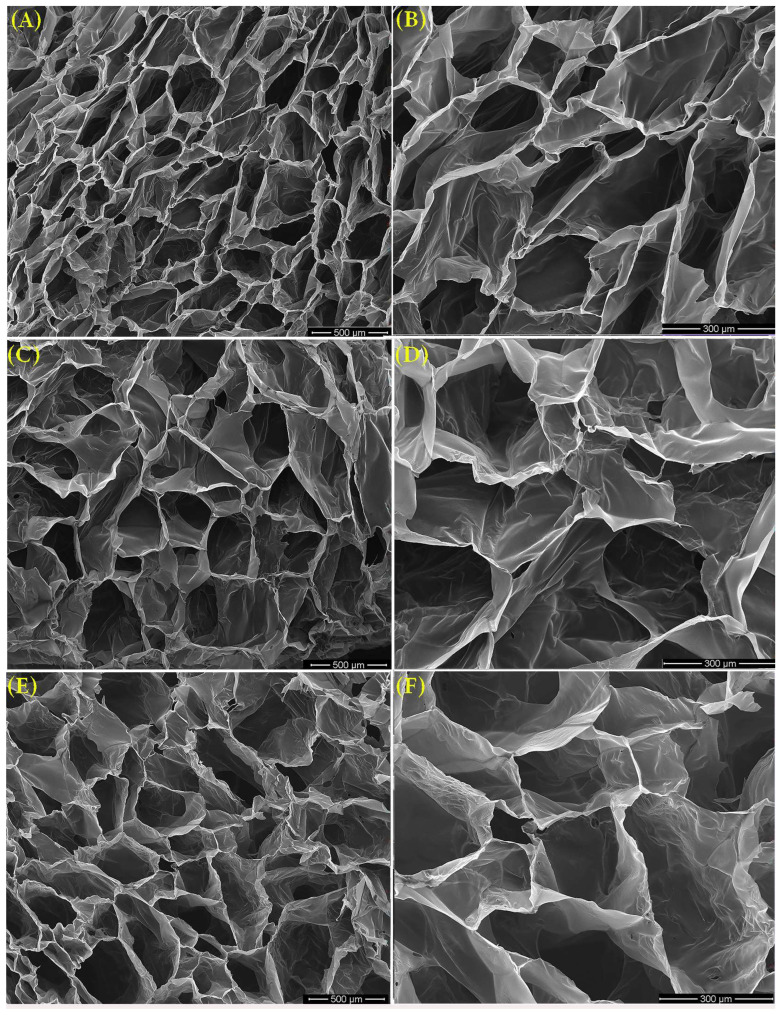
SEM images of LO-enriched hydrogels: (**A**) 5-4, (**C**) 6-4, (**E**) 7-4 at 100× magnification and (**B**) 5-4, (**D**) 6-4, (**F**) 7-4 at 250× magnification.

In contrast, the LO-hydrogels presented a spongiform porous architecture, as exemplified for hydrogels containing Smix 4 (Figure 11), with pore sizes increasing proportionally to LO concentration. The macropores observed in hydrogels were separated by thinner walls, exhibiting a continuous, unbroken structure. This integrity likely contributes to the structural stability of the hydrogel while enhancing elasticity. Such structural features are desirable for wound dressings where flexibility and swelling capacity are necessary [40].

The incorporation of Tween 80 facilitated the formation of a mesh-like structure with interconnected pores. This is likely due to molecular interactions between the functional groups of the surfactant and polymer matrix. The hydroxyl and carbonyl groups of Tween 80 may interact with CMC, PEO, and PVP, forming hydrogen bonds, and contributing to the formation of the hydrogel network. Moreover, hydrophobic interactions between the acetyl groups of CMC and the apolar residues of surfactant molecules inhibited the formation of a dense structure. Instead, these interactions resulted in a softer and more flexible network, enhancing the hydrogel’s ability to swell while preserving its structural integrity [41]. A similar study presented the PVA/Kaolin/Cedar Oil sponge hydrogels, which exhibited a well-defined porous structure with pronounced lamellar formations. The incorporation of cedar oil and kaolin into the PVA matrix led to an increase in pore size within the produced sponges [42].

## 4. Conclusions

This study successfully demonstrated the synthesis of lavender oil-enriched hydrogels using e-beam irradiation and their characterisation, with particular attention to the effects of LO and Tween 80 on the structural and mechanical properties of the hydrogels. 

The results indicated that LO-enriched hydrogels exhibited swelling capacities of 7700% to 18,000%, reaching equilibrium in 10 h for Smix 4 and Smix 5, and in 20 h for Smix 3. Compared to control hydrogel, LO-hydrogels demonstrated enhanced mesh sizes (ranging from 156 nm to 246 nm), adequate elastic moduli (from 130 Pa to 308 Pa for Smix 5), and greater flexibility, suggesting that the incorporation of LO and surfactants can tailor the network structure to enhance hydrophilicity and mechanical adaptability. Among the studied hydrogels, only certain formulations, including Smix 4 and Smix 5, exhibited higher crosslinking density and greater structural stability, highlighting their potential for biomedical applications that require durability and structural integrity. These results indicated that LO:Smix ratio directly impacted the hydrogel features and can be further employed to customise the hydrogel properties for various biomedical applications. The porous architecture observed in LO-hydrogels and their ability to swell significantly make them promising candidates for wound care products that require prolonged moisture retention and flexibility. The larger pores (up to 246 nm) and thinner walls seen in the SEM analysis suggest that the hydrogels can absorb significant amounts of exudate, and the flexible network could allow for reduced dressing changes, enhancing patient comfort and acceptance. Additionally, the LO-hydrogels demonstrated an effective balance between structural stability and biodegradability, exhibiting a degradation rate of only 12% after 35 days.

Based on the promising results, future research should explore their performance focusing on the wound healing efficacy, biocompatibility, and the controlled release of essential oil to validate their suitability for biomedical applications.

## Figures and Tables

**Table 1:** pH, visual appearance, and phase separation after 48 h and 60 days for LO-pre-hydrogels.

Code	pH	Irradiation Dose (kGy)	Appearance	Phase Separation –After 48 h	Phase Separation –After 60 Days
**Smix 3**					
1-3	6.0	70	semi-transparent	no	no
2-3	5.9	70	semi-transparent	no	no
3-3	5.9	70	semi-transparent	no	no
4-3	5.9	70	milky-white	no	no
5-3	6.0	70	milky-white	no	yes
6-3	5.9	70	milky-white	yes	yes
7-3	5.9	70	milky-white	yes	yes
8-3	5.9	70	milky-white	yes	yes
9-3	5.8	70	milky-white	yes	yes
10-3	5.7	70	milky-white	yes	yes
11-3	5.8	70	milky-white	yes	yes
**Smix 4**					
1-4	6.3	30	transparent	no	no
2-4	6.3	30	transparent	no	no
3-4	6.4	30	semi-transparent	no	no
4-4	6.3	30	semi-transparent	no	no
5-4	6.3	30	semi-transparent	no	no
6-4	6.3	30	semi-transparent	no	no
7-4	6.2	30	transparent	no	no
8-4	6.3	30	semi-transparent	no	no
9-4	6.3	30	semi-transparent	no	no
10-4	6.3	30	milky-white	no	yes
11-4	6.2	30	milky-white	yes	yes
**Smix 5**					
1-5	6.6	30	transparent	no	no
2-5	6.6	30	transparent	no	no
3-5	6.6	30	transparent	no	no
4-5	6.6	30	semi-transparent	no	no
5-5	6.7	30	semi-transparent	no	no
6-5	6.6	30	semi-transparent	no	no
7-5	6.6	30	transparent	no	no
8-5	6.5	30	semi-transparent	no	no
9-5	6.6	30	milky-white	no	yes
10-5	6.4	30	milky-white	yes	yes
11-5	6.5	30	milky-white	yes	yes

**Table 2:** Structural parameters of LO-enriched hydrogels.

Samples Code		*G′* (Pa)	*ρ* (g/cm^3^)	$M_{c}$ × 10^4^ (g/mol)	$V_{e}$ × 10^−6^ (mol/cm^3^)	*ξ* (nm)
**Hydrogel**		2169	0.8996	4.07	22.06	43
**Smix 4**	5-4	200	0.9365	40.84	2.29	196
	6-4	175	0.9168	44.18	2.08	204
	7-4	134	1.0010	64.03	1.56	246
**Smix 5**	3-5	219	0.9425	41.81	2.25	184
	4-5	308	0.9842	28.43	3.41	156
	5-5	130	0.9685	78.26	1.23	240

## Data Availability

The data presented in this study are available on request from the corresponding author.

## Supplementary Materials

The following supporting information can be downloaded at: https://www.mdpi.com/article/10.3390/polym16223150/s1; Figure S1: UV–Vis spectra for: (A) Smix 3; (B) Smix 4; (C) Smix 5 after 24 h and (D) Smix 3; (E) Smix 4; (F) Smix 5 after 80 days; Table S1: The Ostwald de Waele model’s rheological parameters and apparent viscosity for Smix 3 samples; Table S2: The Ostwald de Waele model’s rheological parameters and apparent viscosity for Smix 4 samples; Table S3: The Ostwald de Waele model’s rheological parameters and apparent viscosity for Smix 5 samples; Table S4: The values of the parameters in the CIE *L*a*b** coordinate system for Smix 3 samples; Table S5: The values of the parameters in the CIE *L*a*b** coordinate system for Smix 4 samples; Table S6: The values of the parameters in the CIE *L*a*b** coordinate system for Smix 5 samples; Table S7: The values of the parameters in the CIE *L*C*h*^0^ coordinate system for Smix 3 samples; Table S8: The values of the parameters in the CIE *L*C*h*^0^ coordinate system for Smix 4 samples; Table S9: The values of the parameters in the CIE *L*C*h*^0^ coordinate system for Smix 5 samples [43,44,45,46,47].
