# Supplementary material for: Formulation, E-Beam Crosslinking, and Comprehensive Characterisation of Lavender Oil-Enriched Hydrogels
Source: Polymers (Basel). 2024 Nov 12;16(22):3150. doi: 10.3390/polym16223150 (PMC11597954; doi:10.3390/polym16223150)
Supplement: Supplementary file 1 [file polymers-16-03150-s001.zip › polymers-3298711-supplementary.pdf]

Formulation, E-Beam Crosslinking, and Comprehensive Characterisation of Lavender Oil-Enriched Hydrogels

Maria Demeter [1], Ion Călina [1,*], Anca Scărișoreanu [1,*], Monica R. Nemțanu [1], Mirela Brașoveanu [1], Marin Micutz [2] and Marius Dumitru [1]

[1] National Institute for Laser, Plasma and Radiation Physics, 409 Atomiștilor, 077125 Măgurele, Romania; maria.demeter@inflpr.ro (M.D.); monica.nemtanu@inflpr.ro (M.R.N.); mirela.brasoveanu@inflpr.ro (M.B.); marius.dumitru@inflpr.ro (M.D.)
[2] Department of Physical Chemistry, University of Bucharest, 4-12 Regina Elisabeta Blvd, 030018 Bucharest, Romania; marin.micut@chimie.unibuc.ro
* Correspondence: calina.cosmin@inflpr.ro (I.C.); anca.scarisoreanu@inflpr.ro (A.S.)

Table S1. The Ostwald de Waele model's rheological parameters and apparent viscosity for Smix 3 samples.

Sample	k [mPa·s^n]	n	$\eta_a^{200s^{-1}}$ [mPa·s]
IPC	432.8±17.9[a]	0.736±0.006[b]	106.8±1.2[a]
1-3	286.8±7.2[c]	0.664±0.002[a]	48.4±0.6[d]
2-3	204.9±9.3[d]	0.669±0.003[a]	35. 5±1.1[e]
3-3	270.3±5.8[c]	0.778±0.003[d]	83.2±0.4[c]
4-3	352.1±29.3[b]	0.749±0.010c	93.1±2.7[b]

Values in each column with distinct superscripts differ significantly ($p \le 0.05$).

Table S2. The Ostwald de Waele model's rheological parameters and apparent viscosity for Smix 4 samples.

Sample	k [mPa·s^n]	n	$\eta_a^{200s^{-1}}$ [mPa·s]
IPC	432.8±17.9[b]	0.736±0.006[a]	106.8±1.2[c]
1-4	412.7±9.0[bc]	0.743±0.003[ab]	105.7±0.8[cd]
2-4	465.4±12.8[a]	0.747±0.003[bcd]	121.5±5.0[a]
3-4	345.0±8.9[f]	0.748±0.004[bcde]	90.9±0.6[f]
4-4	431.0±22.5[bc]	0.749±0.001[bcde]	114.0±5.6[b]
5-4	378.8±10.7[e]	0.752±0.004[cdef]	101.9±0.5[de]
6-4	405.7±7.8[cd]	0.745±0.003[bc]	104.9±0.2[cde]
7-4	383.5±19.5[de]	0.753±0.006[def]	103.4±1.8[cde]
8-4	369.8±4.0[ef]	0.754±0.001[ef]	100.6±0.6[e]
9-4	345.0±23.3[f]	0.757±0.008[f]	95.2±2.2[f]

Values in each column with distinct superscripts differ significantly ($p \le 0.05$).

Table S3. The Ostwald de Waele model's rheological parameters and apparent viscosity for Smix 5 samples.

Sample	k [mPa·s^n]	n	$\eta_a^{200s^{-1}}$ [mPa·s]
IPC	432.8±17.9[bc]	0.736±0.006[ab]	106.8±1.2[cd]
1-5	479.1±17.8[a]	0.727±0.004[a]	112.5±1.6[a]
2-5	407.2±3.2[cde]	0.741±0.001[bc]	103.0±0.1[ef]
3-5	380.8±13.4[e]	0.750±0.005[cd]	101.0±1.1[f]
4-5	448.3±35.6[ab]	0.736±0.010[ab]	110.4±3.2[ab]
5-5	388.8±14.5[de]	0.748±0.005[cd]	102.4±0.9[ef]
6-5	418.5±27.4[bcd]	0.745±0.008[bcd]	108.1±2.6[bc]
7-5	424.7±7.8[bc]	0.738±0.005[b]	106.1±0.9[cd]
8-5	390.3±15.0[de]	0.751±0.005[d]	104.2±1.1[de]

Values in each column with distinct superscripts differ significantly ($p \le 0.05$).

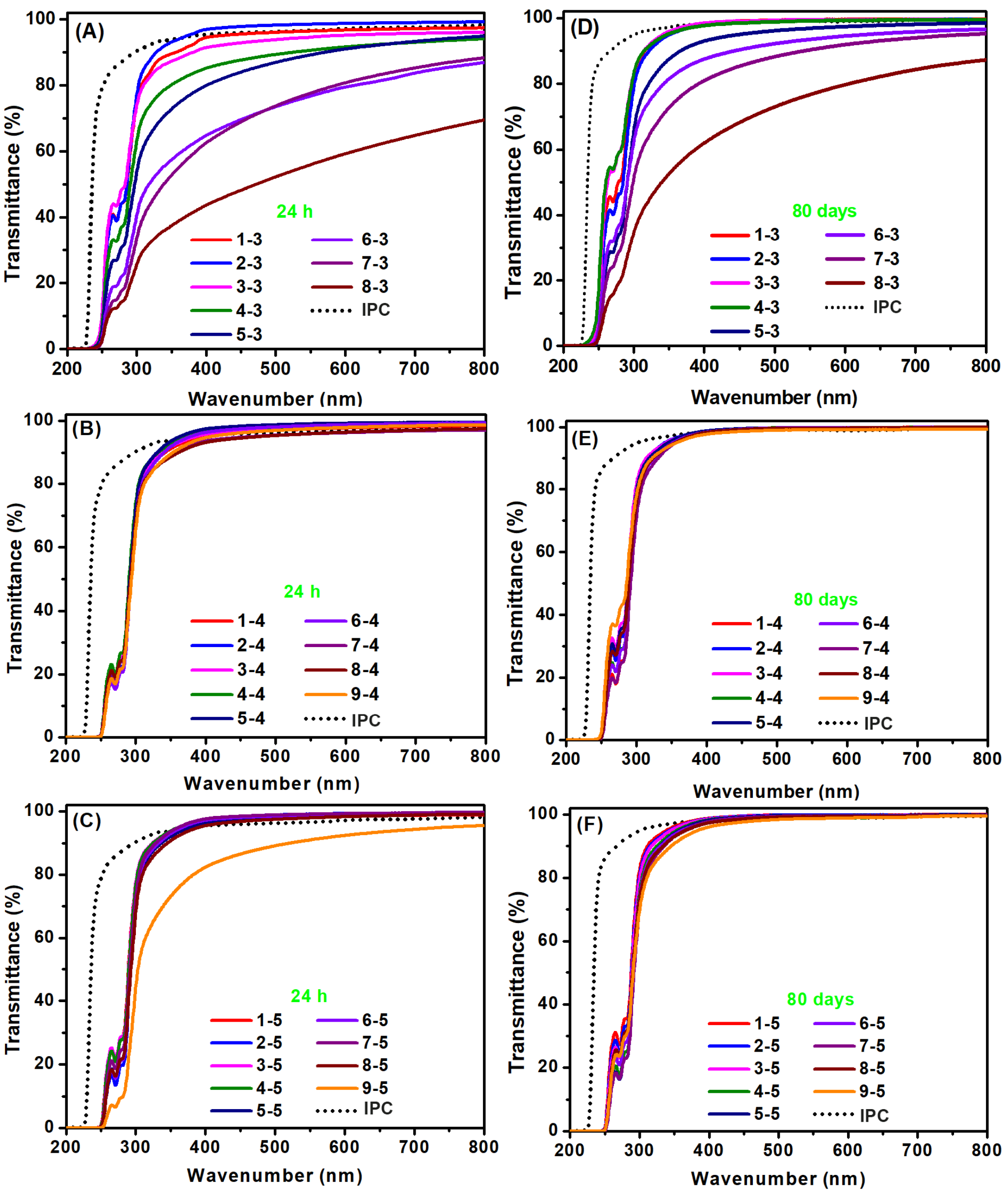

Figure S1. UV–Vis spectra for: (A) Smix 3; (B) Smix 4; (C) Smix 5 after 24 h and (D) Smix 3; (E) Smix 4; (F) Smix 5 after 80 days.

3.3 Colour characterization of pre-hydrogels

Colour is an important element in the visual assessment of materials, playing a significant role in the perceived quality of a product or formulation [1]. Colour variations can provide valuable information regarding changes in a material's structure, composition, or properties, making it a useful quality index [2].

In this study, the CIE $L^*a^*b^*$ and CIE $L^*C^*h^0$ colour spaces were used to evaluate the colour characteristics of the IPC and LO-enriched pre-hydrogels. Based on $L^*a^*b^*$ colour coordinates, the IPC exhibited a nearly neutral colour with a slight yellowish tint, consisting of yellow ($+b^*$) and green ($-a^*$) components along with high lightness (L^*~99%). **Tables S4-S6** show the colour parameter values for each sample. LO-pre-hydrogels containing Smix 3 showed decreases ($p \leq 0.05$) in lightness, indicating increased opacity compared to the IPC. Among these, sample (1-3) exhibited the most substantial reduction in lightness (up to 67%), while sample (3-3) showed only a small decrease (~4%). Conversely, formulations containing Smix 4 and Smix 5 maintained lightness values close to the IPC, with only some formulations, such as (8-4) and (9-4), exhibiting notable reductions of ~11% and ~29%, respectively.

The green (–a^*) and yellow (+b^*) components also showed changes ($p \leq 0.05$) depending on the sample composition. LO-pre-hydrogels with Smix 3 exhibited a shift toward to the red component (+a^*) for all samples, except for sample (3-3). In contrast, most Smix 4 and Smix 5 formulations showed an increase in the green component (–a^*), except for sample (9-4), which displayed the smallest increase, and sample 8-4, which showed a notable shift towards red (+a^*). For the yellow component (+b^*), all LO-pre-hydrogels demonstrated significant increases ($p \leq 0.05$) compared to the IPC. Smix 3 formulations had the highest values overall, while Smix 4 formulations displayed the most pronounced changes in the $L^*a^*b^*$ colour space parameters.

The total colour difference (ΔE_{ab}), a key indicator of overall changes in structure and physical properties [2], showed reflected the shifts ($p \leq 0.05$) in the colour parameters of each sample. Literature [3–5] suggests that human perception of colour differences can be categorised as imperceptible ($\Delta E_{ab} < 1$), slightly perceptible (ΔE_{ab} = 1 – 3), depending on the hue, and easily perceptible ($\Delta E_{ab} > 3$). According to these thresholds, most Smix 3 samples showed perceptible colour changes with high ΔE_{ab} values (ΔE_{ab} = 4.8 – 17.3), indicating notable structural changes and potentially reduced stability. These changes might be linked to phase separation or alterations in the formulation's uniformity over time. In contrast, most Smix 4 and Smix 5 formulations showed only slightly perceptible changes, suggesting better colour stability. However, samples (8-4) and (9-4) demonstrated substantial significant overall changes in structure and composition, reflected in high ΔE_{ab} values ($\Delta E_{ab} > 13.6$), primarily due to changes in L^* and the proportion of a^*. Therefore, formulations with the lowest colour change are more likely to remain stable, while those with greater changes ($\Delta E_{ab} > 5$) may be less stable.

Table S4. The values of the parameters in the CIE $L^*a^*b^*$ coordinate system for Smix 3 samples.

Sample	L^*	a^*	b^*	ΔE_{ab}
IPC	99.56±0.03^{a}	-0.19±0.01^{a}	1.01±0.02^{a}	0.00^{a}
1-3	32.75±1.38^{e}	4.29±0.35^{c}	9.55±0.81^{c}	67.50±1.24^{e}
2-3	37.07±1.27^{d}	5.73±0.14^{d}	13.35±0.68^{d}	63.97±1.10^{d}
3-3	95.66±0.00^{b}	0.00±0.00^{a}	4.81±0.00^{b}	5.45±0.00^{b}
4-3	74.46±0.02^{c}	2.82±0.06^{b}	17.29±0.01^{e}	30.07±0.01^{c}

Values in each column with distinct superscripts differ significantly (p ≤ 0.05).

Table S5. The values of the parameters in the CIE $L^*a^*b^*$ coordinate system for Smix 4 samples.

Sample	L^*	a^*	b^*	ΔE_{ab}
IPC	99.56±0.03^{a}	-0.19±0.01^{b}	1.01±0.02^{a}	0.00^{a}
1-4	99.10±0.01^{ab}	-0.55±0.00^{de}	3.44±0.02^{e}	2.51±0.02^{c}
2-4	98.11±0.02^{c}	-0.59±0.00^{f}	4.47±0.03^{h}	3.78±0.03^{d}
3-4	99.04±0.01^{b}	-0.53±0.00^{d}	3.27±0.02^{c}	2.35±0.02^{bc}
4-4	99.03±0.01^{b}	-0.54±0.00^{de}	3.36±0.02^{d}	2.43±0.02^{bc}
5-4	99.56±0.01^{a}	-0.53±0.06^{de}	3.00±0.01^{b}	2.03±0.02^{b}
6-4	99.51±0.00^{a}	-0.59±0.01^{f}	3.26±0.01^{c}	2.29±0.01^{bc}
7-4	98.88±0.00^{b}	-0.57±0.00^{ef}	3.56±0.01^{f}	2.67±0.01^{c}
8-4	88.35±0.04^{d}	0.60±0.01^{a}	8.78±0.02^{i}	13.66±0.04^{e}
9-4	99.56±0.03^{a}	-0.19±0.01^{b}	1.01±0.02^{a}	0.00^{a}

Values in each column with distinct superscripts differ significantly (p ≤ 0.05).

Table S6. The values of the parameters in the CIE $L^*a^*b^*$ coordinate system for Smix 5 samples.

Sample	L^*	a^*	b^*	ΔE_{ab}
IPC	99.56±0.03^{a}	-0.19±0.01^{a}	1.01±0.02^{a}	0.00^{a}
1-5	98.47±0.67^{d}	-0.53±0.10^{c}	3.52±0.16^{e}	2.80±0.37^{d}
2-5	99.17±0.08^{abc}	-0.54±0.00^{c}	3.34±0.03^{cd}	2.39±0.03^{bc}
3-5	99.26±0.38^{ab}	-0.53±0.01^{c}	3.20±0.05^{b}	2.26±0.05^{b}
4-5	98.77±0.22^{cd}	-0.54±0.06^{c}	3.29±0.02^{bc}	2.45±0.08^{bc}
5-5	98.98±0.25^{bc}	-0.56±0.03^{cd}	3.44±0.16^{de}	2.53±0.15^{c}
6-5	99.27±0.01^{ab}	-0.62±0.01^{d}	3.79±0.00^{f}	2.83±0.00^{d}
7-5	99.19±0.01^{abc}	-0.59±0.00^{cd}	3.77±0.01^{f}	2.82±0.01^{d}
8-5	97.11±0.03^{e}	-0.41±0.02^{b}	5.13±0.03^{g}	4.80±0.02^{e}

Values in each column with distinct superscripts differ significantly (p ≤ 0.05).

The CIE $L^*C^*h^0$ colour space analysis further confirmed these findings. In the CIE $L^*C^*h^0$ colour space, lightness L^* remains similar to that in the CIE $L^*a^*b^*$ system, chroma C^* represents colour saturation, and hue angle h^0 indicates the qualitative colour, expressed in degrees: 0° (red), 90° (yellow), 180° (green), and 270° (blue). For the IPC, the chroma and hue values were 1.02±0.02 and 100.96±0.32°, respectively, with a lightness of 99.56±0.03%, corresponding to a relatively neutral but clear yellow-greenish colour in the CIE $L^*a^*b^*$ space. **Tables S7-S9** show the colour parameters for each investigated sample. Saturation showed a significant increase ($p \leq 0.05$) for all Smix 3 formulations, particularly for samples (1-3), (2-3), and (4-3), where C^* exceeded 10. On the other hand, the hue showed a slight downward shift ($p \leq 0.05$) from the IPC, indicating a transition toward yellow for most samples, except for formulations (1-3), (2-3), and (4-3), which shifted toward red.

Table S7. The values of the parameters in the CIE $L^*C^*h^0$ coordinate system for Smix 3 samples.

**Sample**	L^*	C^*	h°
IPC	$99.56±0.03^a$	$1.02±0.02^a$	$100.96±0.32^a$
1-3	$32.75±1.38^e$	$10.47±0.88^c$	$65.81±0.05^e$
2-3	$37.07±1.27^d$	$14.53±0.67^d$	$66.76±0.57^d$
3-3	$95.66±0.00^b$	$4.81±0.00^b$	$90.05±0.07^b$
4-3	$74.46±0.02^c$	$17.52±0.02^e$	$80.75±0.20^c$

* *Values in each column with distinct superscripts differ significantly ($p \leq 0.05$).*

Table S8. The values of the parameters in the CIE $L^*C^*h^0$ coordinate system for Smix 4 samples.

**Sample**	L^*	C^*	h°
IPC	$99.56±0.03^a$	$1.02±0.02^a$	$100.96±0.32^a$
1-4	$99.10±0.01^{ab}$	$3.49±0.02^e$	$99.00±0.10^c$
2-4	$98.11±0.02^c$	$4.51±0.03^h$	$97.54±0.06^d$
3-4	$99.04±0.01^b$	$3.31±0.02^c$	$99.13±0.04^c$
4-4	$99.03±0.01^b$	$3.40±0.02^d$	$99.18±0.11^c$
5-4	$99.56±0.01^a$	$3.04±0.02^b$	$100.09±1.11^b$
6-4	$99.51±0.00^a$	$3.32±0.01^c$	$100.22±0.10^b$
7-4	$98.88±0.00^b$	$3.60±0.01^f$	$99.08±0.01^c$
8-4	$88.35±0.04^d$	$8.80±0.02^i$	$86.09±0.05^f$
9-4	$70.86±0.04^e$	$3.70±0.06^g$	$93.94±0.44^e$

* *Values in each column with distinct superscripts differ significantly ($p \leq 0.05$).*

Table S9. The values of the parameters in the CIE $L^*C^*h^0$ coordinate system for Smix 5 samples.

**Sample**	L^*	C^*	h°
IPC	$99.56±0.03^a$	$1.02±0.02^a$	$100.96±0.32^a$
1-5	$98.47±0.67^d$	$3.56±0.14^e$	$98.65±1.95^b$
2-5	$99.17±0.08^{abc}$	$3.38±0.03^{cd}$	$99.13±0.04^b$
3-5	$99.26±0.38^{ab}$	$3.24±0.05^b$	$99.38±0.30^b$
4-5	$98.77±0.22^{cd}$	$3.34±0.02^{bc}$	$99.40±0.98^b$
5-5	$98.98±0.25^{bc}$	$3.48±0.15^{de}$	$99.32±0.95^b$
6-5	$99.27±0.01^{ab}$	$3.84±0.00^f$	$99.22±0.09^b$
7-5	$99.19±0.01^{abc}$	$3.82±0.01^f$	$98.90±0.09^b$
8-5	$97.11±0.03^e$	$5.14±0.03^g$	$94.58±0.25^c$

* *Values in each column with distinct superscripts differ significantly ($p \leq 0.05$).*

References

1. Nemtanu, M.; Brasoveanu, M. Functional Properties of Some Non-Conventional Treated Starches. In *Biopolymers*; Elnashar, M., Ed.; IntechOpen: Rijeka, 2010; pp. 319–344.

2. Nemţanu, M.R.; Braşoveanu, M. Impact of Electron Beam Irradiation on Quality of Sea Buckthorn (*Hippophae Rhamnoides* L.) Oil. *J Sci Food Agric* **2016**, *96*, 1736–1744, https://doi:10.1002/jsfa.7280.

3. Bodart, M.; de Peñaranda, R.; Deneyer, A.; Flamant, G. Photometry and Colorimetry Characterisation of Materials in Daylighting Evaluation Tools. *Build Environ* **2008**, *43*, 2046–2058. https://doi.org/10.1016/j.buildenv.2007.12.006.

4. Castagna, A.; Chiavaro, E.; Dall'Asta, C.; Rinaldi, M.; Galaverna, G.; Ranieri, A. Effect of Postharvest UV-B Irradiation on Nutraceutical Quality and Physical Properties of Tomato Fruits. *Food Chem* **2013**, *137*, 151–158. https://doi.org/10.1016/j.foodchem.2012.09.095.

5. Sant'Anna, V.; Gurak, P.D.; Ferreira Marczak, L.D.; Tessaro, I.C. Tracking Bioactive Compounds with Colour Changes in Foods – A Review. *Dyes and Pigments* **2013**, *98*, 601–608. https://doi.org/10.1016/j.dyepig.2013.04.011.
